# Single-Nucleotide Polymorphisms of the *PAR2* and *IL-17A* Genes Are Significantly Associated with Chronic Pain

**DOI:** 10.3390/ijms242417627

**Published:** 2023-12-18

**Authors:** Moe Soeda, Seii Ohka, Daisuke Nishizawa, Masako Iseki, Keisuke Yamaguchi, Hideko Arita, Kazuo Hanaoka, Jitsu Kato, Setsuro Ogawa, Ayako Hiranuma, Junko Hasegawa, Kyoko Nakayama, Yuko Ebata, Masakazu Hayashida, Tatsuya Ichinohe, Ken-ichi Fukuda, Kazutaka Ikeda

**Affiliations:** 1 Addictive Substance Project, Tokyo Metropolitan Institute of Medical Science, Tokyo 156-8506, Japan; soedamoe@tdc.ac.jp (M.S.); ohka-si@igakuken.or.jp (S.O.); nishizawa-ds@igakuken.or.jp (D.N.);; 2 Department of Oral Health and Clinical Science, Tokyo Dental College, Tokyo 101-0061, Japan; 3 Department of Anesthesiology & Pain Medicine, Juntendo University School of Medicine, Tokyo 113-8431, Japan; miseki@juntendo.ac.jp (M.I.);; 4 Department of Anesthesiology, Pain Relief Center, JR Tokyo General Hospital, Tokyo 151-8528, Japan; h-arita-komaba@hotmail.co.jp (H.A.); hanaokajre@gmail.com (K.H.); 5 Department of Anesthesiology, Nihon University School of Medicine, Tokyo 173-8610, Japan; 6 University Research Center, Nihon University, Tokyo 173-8610, Japan; 7 Department of Surgery, Toho University Sakura Medical Center, Chiba 285-8741, Japan; 8 Department of Anesthesiology, Saitama Medical University International Medical Center, Saitama 350-1298, Japan; 9 Department of Dental Anesthesiology, Tokyo Dental College, Tokyo 101-0061, Japan; ichinohe@tdc.ac.jp

**Keywords:** chronic pain, protease-activated receptor 2, interleukin 17A, T-helper 17, astrocyte

## Abstract

Patients with chronic pain are affected psychologically and socially. There are also individual differences in treatment efficacy. Insufficient research has been conducted on genetic polymorphisms that are related to individual differences in the susceptibility to chronic pain. Autoimmune disorders can lead to inflammation and chronic pain; therefore, we focused on the autoimmune-related protease-activated receptor 2 (*PAR2*/*F2RL1*) and interleukin 17A (*IL-17A*/*IL17A*) genes. PAR2 and IL-17A are associated with autoimmune diseases that lead to chronic pain, and PAR2 regulates T-helper (Th) cell activation and differentiation. We hypothesized that the *PAR2* and *IL-17A* genes are associated with chronic pain. The present study used a case–control design to statistically examine associations between genetic polymorphisms and the vulnerability to chronic pain. The rs2243057 polymorphism of the *PAR2* gene and rs3819025 polymorphism of the *IL-17A* gene were previously reported to be associated with pain- or autoimmune-related phenotypes. Thus, these polymorphisms were investigated in the present study. We found that both rs2243057 and rs3819025 were significantly associated with a susceptibility to chronic pain. The present findings revealed autoimmune-related genetic factors that are involved in individual differences in chronic pain, further aiding understanding of the pathomechanism that underlies chronic pain and possibly contributing to future personalized medicine.

## 1. Introduction

The prevalence of chronic pain in Japan is estimated to be ~30%. Chronic pain patients are affected by many psychological and social factors [1]. Various medications are used to treat chronic pain, but their effectiveness varies from person to person, and adequate treatment is often a challenge. Although associations between chronic pain and genetic polymorphisms have been reported [2,3], insufficient research has been conducted on genetic polymorphisms that are related to individual differences in the susceptibility to chronic pain and the efficacy of therapeutic drugs. The pathogenic mechanism of chronic pain is also not fully understood. 

Protease-activated receptor 2 (PAR2/F2RL1) is a member of the G protein-coupled seven transmembrane receptor family [4,5,6] and is abundantly expressed by most cell types, including immune cells [7,8]. It is also present in neurons in the human central nervous system, including astrocytes, microglia, central terminals of primary afferent nerve fibers, and dorsal horn neurons [5,6,9,10]. PAR2 has been reported to be associated with pain, including mechanical allodynia and mechanical hyperalgesia in cancer and irritable bowel syndrome patients [5,9,11,12,13]. 

T lymphocytes play a central role in chronic neuropathic pain [14]. T-helper 17 (Th17) cells and astrocytes in the central nervous system express functional interleukin 17A (IL-17A) receptors [15]. IL-17A is a cytokine that is secreted by cells of an innate lineage, including Th17 cells [4,16]. Excess IL-17A is associated with abnormal inflammation and implicated in rheumatoid arthritis, asthma, systemic lupus erythematosus, and irritable bowel syndrome [16,17]. Th cells that express IL-17A are associated with pain, including hypersensitivity to mechanical pain [18], and are involved in the pathology of chronic pain and pain intensity [19]. Th17 and IL-17A are also associated with the severity and progression of autoimmune diseases [15,20]. PAR2 regulates the activation and differentiation of Th cells that produce IL-17A [4,21,22]. 

IL-17A impairs the blood–brain barrier integrity, which allows Th17 cells and IL-17A to migrate toward the central nervous system parenchyma [23,24]. IL-17A also activates astrocytes, resulting in the progression of neuroinflammation [15,23,25]. Furthermore, nerve injury promotes neuroinflammation in a PAR2-dependent manner in astrocytes [26]. Both IL-17A and PAR2 are associated with chronic pain [5,9,11,12,13,18,19], and chronic pain can be caused by neuroinflammation that results from activated astrocytes through pathways that involve PAR2 and IL-17A. However, direct evidence of such pathways for chronic pain that involve both PAR2 and IL-17A has not yet been demonstrated.

We previously proposed that the rs2243057 single-nucleotide polymorphism (SNP) of the *PAR2* gene is associated with cold pain sensitivity [27], but no study has reported an association between the rs2243057 SNP of *PAR2* and chronic pain. We hypothesized that the autoimmune-related *IL-17A* gene is also associated with chronic pain. The rs3819025 SNP of *IL-17A* was reported to be associated with autoimmune disease [28]. Therefore, we focused on the rs3819025 SNP of the *IL-17A* gene. In the present study, SNPs of the *PAR2* and *IL-17A* genes were analyzed to explore genetic factors of chronic pain, and we mined public genetic and/or gene expression data to predict logical relationships among SNPs, chronic pain, and gene expression. The results showed that the rs2243057 SNP of *PAR2* and rs3819025 SNP of *IL-17A* were significantly associated with a susceptibility to chronic pain. Our finding that a higher PAR2 expression is related to a susceptibility to chronic pain, together with the public databases and previous reports, indicates that PAR2 is involved in the production of IL-17A by Th17 cells and astrocytes, resulting in chronic pain.

## 2. Results

### 2.1. rs2243057 SNP of PAR2 Gene Is Associated with Chronic Pain

**Figure 1:**
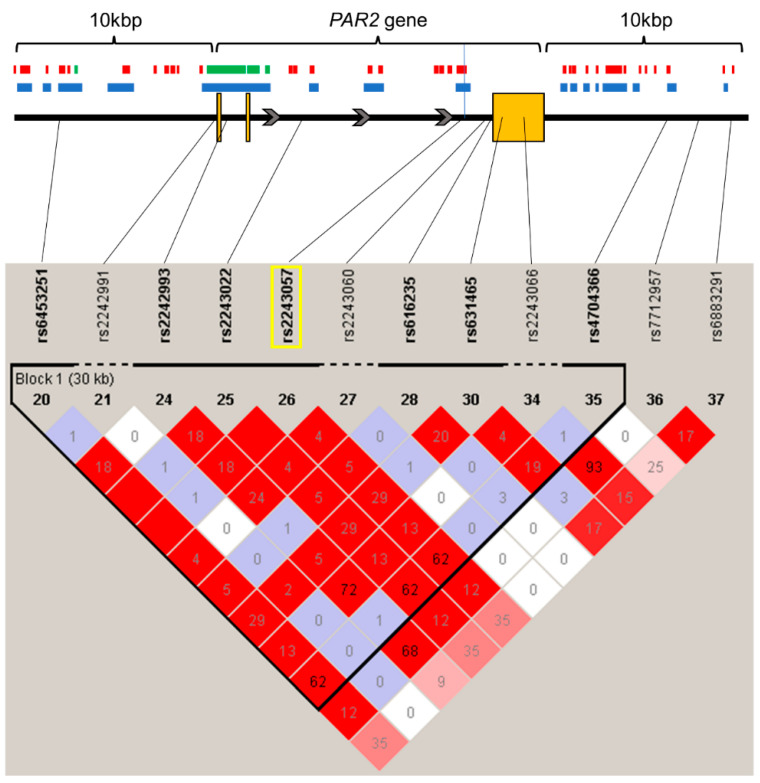
State of LD among SNPs in the *PAR2* gene region, including 10 kbp upstream and downstream (LD Plot-*r*^2^). Numbers in squares that two SNPs face represent the percentage of *r*^2^ values that were calculated from genotype data of the SNPs. The white boxes represent *D*′ < 1 and log of the likelihood odds ratio (LOD) < 2. The shades of pink or red boxes represent *D*′ < 1 and LOD ≥ 2. The blue boxes represent *D*′ = 1 and LOD < 2. The bright red boxes represent *D*′ = 1 and LOD ≥ 2. The solid horizontal line above the LD plot represents the *PAR2* gene, including 10 kbp upstream and downstream of the gene. The orange boxes represent exons, and solid lines represent untranslated regions or introns in the *PAR2* gene structure. The gray arrows represent the direction of transcription. The yellow rectangle represents the SNP on which we focused in this study. The red and green boxes represent the enhancer and promoter regions, respectively, by DHS. The blue boxes represent open chromatin regions with DNase-Seq signals (*p*-value signal ≥ 2). The black vertical line represents the location of the rs2243057 SNP.

Associations between the intronic rs2243057 SNP of the *PAR2* gene and chronic pain were analyzed in 194 adult patients who suffered from chronic pain and 282 adult healthy controls who had no particular diseases, including chronic pain. As shown in Figure 1, the LD analysis for SNPs within and around the *PAR2* gene, including the rs2243057 SNP, showed that the rs2243057 SNP was in complete LD with the rs6453251 and rs2243022 SNPs.

The patients’ genotype distributions of the rs2243057 SNP are shown in Table 1.

The rs2243057 SNP of *PAR2* did not deviate from the theoretical Hardy–Weinberg equilibrium (*p* = 0.26, *χ*^2^ = 1.835). As shown in a previous study, these data had no bias in distribution [28]. Pearson’s *χ*^2^ test revealed a significant difference between the AA group and AG + GG group for patients with chronic pain and healthy controls, suggesting that the rs2243057 SNP is significantly associated with chronic pain in our samples (*p* = 0.038; Table 1). The rates of patients with the AA genotype of the rs2243057 SNP of *PAR2* were lower in patients with chronic pain than in healthy controls (patients: 4.7%; healthy controls: 9.9%; Table 1). Thus, the rate of AG + GG genotypes was higher in patients with chronic pain. This result suggests that G alleles of the rs2243057 SNP of *PAR2* are related to a higher rate of patients with chronic pain. No significant differences in the distribution of this SNP were observed among genotype groups (AA, AG, and GG) or between the AA + AG group and GG group for patients with chronic pain and healthy controls (*p* > 0.05; Table 1). The results suggest that major G alleles of the rs2243057 SNP of *PAR2* are associated with a susceptibility to chronic pain.

### 2.2. rs3819025 SNP of the IL-17A Gene Is Associated with Chronic Pain

**Figure 2:**
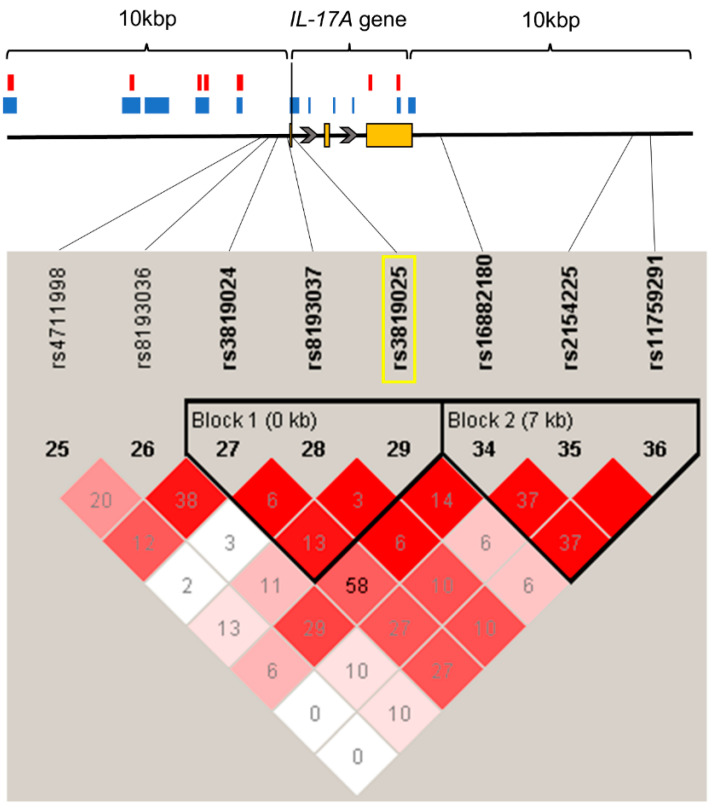
State of LD among SNPs in the *IL-17A* gene region, including 10 kbp upstream and downstream (LD Plot-*r*^2^). Numbers in squares that two SNPs face represent the percentage of *r*^2^ values that were calculated from genotype data of the SNPs. The white boxes represent *D*′ < 1 and log of the likelihood odds ratio (LOD) < 2. The shades of pink or red boxes represent *D*′ < 1 and LOD ≥ 2. The blue boxes represent *D*′ = 1 and LOD < 2. The bright red boxes represent *D*′ = 1 and LOD ≥ 2. The solid horizontal line above the LD plot represents the *IL-17A* gene, including 10 kbp upstream and downstream of the gene. The orange boxes represent exons, and solid lines represent untranslated regions or introns in the *IL-17A* gene structure. The gray arrows represent the direction of transcription. The yellow rectangle represents the SNP on which we focused in this study. The red boxes represent the enhancer regions by DHS. The blue boxes represent open chromatin regions with DNase-Seq signals (*p*-value signal ≥ 2). The black vertical line represents the location of the rs3819025 SNP.

The association between the intronic rs3819025 SNP of *IL-17A* and chronic pain was analyzed in 194 adult patients who suffered from chronic pain and 282 adult healthy controls who had no particular diseases, including chronic pain. The LD analysis of SNPs within and around the *IL-17A* gene, including the rs3819025 SNP, showed that no SNPs were in strong LD with the rs3819025 SNP (Figure 2).

The patients’ genotype distributions of the rs3819025 SNP are shown in Table 2.

The rs3819025 SNP of *IL-17A* did not deviate from the theoretical Hardy–Weinberg equilibrium (*p* = 1, *χ*^2^ = 0.0001). Pearson’s *χ*^2^ test revealed a significant difference among genotype groups (AA, AG, and GG), between the AA group and AG + GG group, and between the AA + AG group and GG group for patients with chronic pain and healthy controls, suggesting that the rs3819025 SNP of IL-17A is significantly associated with chronic pain in our samples (AA vs. AG vs. GG, *p* = 0.016; AA vs. AG + GG, *p* = 0.029; AA + AG vs. GG, *p* = 0.013; Table 2). The rates of patients with the GG genotype of the rs3819025 SNP of *IL-17A* were higher in patients with chronic pain than in healthy controls (patients: 61.3%; healthy controls: 49.6%; Table 2). This result suggests that G alleles of the rs3819025 SNP of *IL-17A* are related to a higher rate of patients with chronic pain. To ascertain linearity of the higher rate of patients by copy number of the G allele of the SNP, we applied the Cochran–Armitage trend test, which revealed a positive correlation between the rate of patients and copy number of the G allele of the rs3819025 SNP (*p* = 0.0042, Cochran–Armitage trend test). The results suggest that major G alleles of the rs3819025 SNP of *IL-17A* are associated with a susceptibility to chronic pain.

## 3. Discussion

The present study suggests that the rs2243057 SNP of *PAR2* and rs3819025 SNP of *IL-17A* are associated with a susceptibility to chronic pain. The results revealed genetic factors that are involved in individual differences in chronic pain, further aiding understanding of the pathomechanism that underlies chronic pain and possibly contributing to future personalized medicine.

Expression quantitative trait loci that were identified by GTEx showed that the amount of *PAR2* mRNA expression that depends on rs2243057 genotypes is AA < AG < GG in brain tissues and the spinal cord (anterior cingulate cortex [BA24], *p* = 1.6 × 10^−11^, m-value = 1; cortex, *p* = 2.0 × 10^−12^, m-value = 1; frontal cortex [BA9], *p* = 8.7 × 10^−14^, m-value = 1; spinal cord [cervical c-1], *p* = 4.3 × 10^−5^, m-value = 1; GTEx Portal), indicating a genetic effect of rs2243057 genotypes on *PAR2* mRNA expression (Figure S1) [29]. These public data indicate that the major G allele of *PAR2* rs2243057 SNP is associated with a higher *PAR2* mRNA expression. Together with our present findings that carriers of the major G allele of the rs2243057 SNP of *PAR2* were significantly more susceptible to chronic pain (Table 1), it is feasible that the chronic pain susceptibility by the major G allele of *PAR2* rs2243057 SNP is associated with a higher *PAR2* mRNA expression.

As shown in Figure 1 and Table S1, the rs2243057 SNP of the *PAR2* gene is located in an open chromatin and enhancer region in ionomycin-stimulated Th17 primary cells and astrocytes [30]. Together with the public data above which show that the major G allele of *PAR2* rs2243057 SNP is associated with a higher *PAR2* mRNA expression, these data suggest that the major G allele of rs2243057 SNP of *PAR2* enhances *PAR2* mRNA and protein expression in astrocytes and ionomycin-stimulated Th17 cells. Thus, the chronic pain susceptibility by the major G allele of *PAR2* rs2243057 SNP could be associated with a higher *PAR2* mRNA expression in astrocytes and ionomycin-stimulated Th17 cells. Furthermore, the rs3819025 SNP of the *IL-17A* gene is located in an open chromatin region in ionomycin-stimulated Th17 primary cells (Figure 2 and Table S1) [31], indicating that the rs3819025 SNP of *IL-17A* would affect *IL-17A* gene expression levels in activated Th17 cells. Although it is unknown whether the G allele of the *IL-17A* rs3819025 SNP is linked to the higher expression of *IL-17A* mRNA in humans in vivo, previous studies may support this possibility. The AA genotype of the *IL-17A* rs2275913 SNP and a haplotype comprising rs2275913 A/rs3819025 G/rs3748067 G of the *IL-17A* gene were associated with a higher risk of viral myocarditis in humans [32]. Moreover, the AA genotype of the *IL-17A* rs2275913 SNP was linked to higher serum IL-17A levels compared with GG/AG genotypes, and these higher serum IL-17A levels correlated with cardiac damage in viral myocarditis patients. Thus, the G allele of the *IL-17A* rs3819025 SNP as an element of the haplotype may be related to at least a higher tendency toward a higher IL-17A expression in combination with the rs2275913 A and rs3748067 G alleles. Together with our findings that carriers of the major G allele of the rs3819025 SNP of *IL-17A* were significantly more susceptible to chronic pain (Table 2) and that the rs3819025 SNP of the *IL-17A* gene is located in an open chromatin region in ionomycin-stimulated Th17 primary cells (Figure 2 and Table S1), the major G allele of the rs3819025 SNP might be associated with high *IL-17A* gene expression levels in activated Th17 cells.

As indicated above, the high expression levels of the PAR2 and IL-17A proteins by the major alleles of the SNPs that were focused on in astrocytes and/or activated Th17 cells could be associated with chronic pain. Together with the fact that PAR2 regulates the activation and differentiation of Th cells that produce IL-17A [4,21,22] and that chronic pain can be caused by neuroinflammation that results from activated astrocytes through a pathway that involves PAR2 and IL-17 (as discussed in the Section 1), our results appear to support the following notion. After nerve injury, (1) PAR2 is upregulated in astrocytes and activated Th17 cells, (2) the upregulation of PAR2 on astrocytes enhances IL-17A expression, (3) the upregulation of PAR2 on activated Th17 cells enhances IL-17A expression and Th cell differentiation, and (4) the expression of IL-17A is further enhanced in activated Th17 cells, thereby resulting in chronic pain, such as pain that is associated with neuroinflammation and neuropathic pain. The SNPs of the *PAR2* and *IL-17A* genes on which we focused in the present study may participate in these pathways, but further research is needed to elucidate the precise mechanism. The inflammatory immune system appears to be essential in this mechanism; thus, it is also important to confirm the expression of PAR2 and IL-17 in astrocytes and Th17 cells that are derived from individuals with autoimmune diseases and the association of SNPs of the *PAR2* and *IL-17A* genes with autoimmune diseases in future research.

A previous study reported that carriers of the minor A allele of the rs2243057 SNP of *PAR2* exhibited a decrease in cold pain sensitivity, indicating that the major G allele of the rs2243057 SNP is associated with an increase in cold pain sensitivity [27]. From an inflammation perspective, this higher sensitivity of carriers of the major G allele of the rs2243057 SNP is consistent with the present results, in which carriers of the major G allele of rs2243057 are more susceptible to chronic pain. A minor variant A allele of rs3819025 of the *IL-17A* gene protects against Graves’ disease, an autoimmune thyroid disease [28]. Our results indicate that carriers of the major G allele of rs3819025 are more susceptible to chronic pain, which is consistent with these Graves’ disease findings.

## 4. Materials and Methods

### 4.1. Design

The present study used a case–control design to examine the impact of the rs2243057 and rs3819025 SNPs on susceptibility to chronic pain in patients with chronic pain and healthy control participants.

### 4.2. Patients with Chronic Pain and Healthy Participants

Enrolled in the study were 194 adult patients with chronic pain who visited JR Tokyo General Hospital (Tokyo, Japan), Juntendo University Hospital (Tokyo, Japan), or Nihon University Itabashi Hospital (Tokyo, Japan) for the treatment of chronic pain during the period from 2008 to 2015. All of the subjects were Japanese. Most of the patients were treated with analgesics before recruitment or were scheduled to be treated with analgesics at recruitment. Patients with severe coexisting complications were excluded. The detailed demographic and clinical data of the subjects are provided in a previous report [2]. Briefly, chronic pain included neck pain, postoperative pain, spinal canal stenosis, intervertebral disk hernia, lower back pain, postherpetic neuralgia, and others in the present study.

As controls in the study, we enrolled 282 adult healthy volunteer participants during the period from 2004 to 2005 who had no particular diseases, including chronic pain, and who lived in or near the Kanto area in Japan. The detailed demographic data of the subjects and their statistics are detailed in previous reports [33,34].

### 4.3. Genotyping and Linkage Disequilibrium Analysis

We examined SNPs of the *PAR2* and *IL-17A* genes. We analyzed 17 and 12 SNPs around the PAR2 and IL-17A gene regions (including 10 kilobase pair [kbp] upstream and downstream), respectively, using genotype data from whole-genome genotyping in 194 patients and 282 healthy volunteer participants. Genomic DNA was extracted from whole-blood samples using standard procedures. The extracted DNA was dissolved in TE buffer (10 mM Tris-HCl and 1 mM ethylenediaminetetraacetic acid, pH 8.0). The DNA concentration was adjusted to 5–50 ng/μL for genotyping the rs2243057 and rs3819025 SNPs or 100 ng/μL for whole-genome genotyping using a NanoDrop ND-1000 Spectrophotometer (Thermo Fisher Scientific K.K., Tokyo, Japan).

To perform real-time polymerase chain reaction with TaqMan probe detection with a LightCycler 480 (Roche Diagnostics K.K., Tokyo, Japan), TaqMan SNP Genotyping Assays (Thermo Fisher Scientific K.K.) were used that included sequence-specific forward and reverse primers to amplify the polymorphic sequence and two probes that were labeled with VIC and FAM dye to detect both alleles of the *PAR2* and *IL-17A* SNPs. The sequences of the primers for rs2243057 and rs3819025 were not disclosed. Real-time polymerase chain reaction was performed in a final volume of 10 μL that contained 2× LightCycler 480 Probes Master (Roche Diagnostics K.K.), a 40× TaqMan SNP Genotyping Assay probe, 5–50 ng genomic DNA as the template, and up to 10 μL H_2_O equipped with 2× LightCycler 480 Probes Master. The thermal conditions were the following: 95 °C for 10 min, followed by 45 cycles of 95 °C for 10 s and 60 °C for 60 s, with final cooling at 50 °C for 30 s. Afterward, endpoint fluorescence was measured for each sample well, and the A/A, A/G, and G/G genotypes were determined based on the presence or absence of each type of fluorescence.

To identify relationships of the SNPs in the study, linkage disequilibrium (LD) analysis was performed using Haploview v. 4.1 [35]. To estimate the LD strength between SNPs, the commonly used *D*′ and *r*^2^ values were pairwise calculated using the genotype dataset of each SNP. Linkage disequilibrium blocks were defined among the SNPs that showed “strong LD” based on the default algorithm of Gabriel et al. [36], in which the upper and lower 95% confidence limits on *D*′ for strong LD were set at 0.98 and 0.7, respectively. TagSNPs in the LD block were then determined using the Tagger software package, which is incorporated in Haploview v. 4.1 and was detailed in a previous report [37].

### 4.4. Public Database Search

We extracted information on expression quantitative trait loci of the rs2243057 SNP using the GTEx Portal [29] to examine effects on gene expression levels in each cell type. We also extracted DNA sequence features plus additional chromatin accessibility (DHS), DNase-Seq signal of the genes’ genomic regions using ZENBU, to investigate the transcriptional regulation around the SNP regions in the genes on 3 July 2023 [30,31]. The data source of DHS was Roadmap Consortiums in 111 samples, showing promoter, enhancer, and dyadic regions. The data source of the DNase-Seq signal was Roadmap Consortiums in 127 samples, showing open chromatin regions only with *p*-value signal ≥ 2. Data on promoters and enhancers were derived from DNase I-accessible regulatory regions that were defined by Roadmap Consortiums. Open chromatin regions correspond to gene expression control regions, such as promoters and enhancers.

### 4.5. Statistical Analysis

The patients’ demographic and clinical data are expressed as the mean ± standard deviation. The statistical analysis was performed using SPSS v. 20 software (IBM Japan, Tokyo, Japan) and Prism 7.00 (GraphPad, San Diego, CA, USA). The *χ*^2^ test and Fisher’s exact test were used for all genotype frequency data to investigate deviations in the distributions from those in theoretical Hardy–Weinberg equilibrium. The Cochran–Armitage trend test was performed for the genotype-based test for associations. In all of the statistical tests, the criterion for significance was set at *p* < 0.05.

## 5. Conclusions

The present study demonstrated that the rs2243057 SNP of *PAR2* and rs3819025 SNP of *IL-17A* are associated with a susceptibility to chronic pain. These SNPs may affect the regulation of IL-17A expression by PAR2, which is involved in astrocyte-mediated chronic pain. Our findings reveal additional genetic factors that are involved in individual differences in chronic pain, further aiding understanding of the pathomechanism that underlies chronic pain and possibly contributing to future personalized medicine.

## Figures and Tables

**Table 1:** Genotype distributions and comparisons of genotype data between patients with chronic pain and healthy subjects of *PAR2* rs2243057 SNP.

Genotype Groups	Attribution	n	n (%)	*p*
AA/AG/GG	Chronic pain	9/80/102	4.7/41.9/53.4	0.079
	Healthy subjects	28/101/153	9.9/35.8/54.3	
AA/AG + GG	Chronic pain	9/182	4.7/95.3	0.038 *
	Healthy subjects	28/254	9.9/90.1	
AA + AG/GG	Chronic pain	89/102	46.6/53.4	0.85
	Healthy subjects	129/153	45.7/54.3	

*: *p* < 0.05.

**Table 2:** Genotype distributions and comparisons of genotype data between patients with chronic pain and healthy subjects of *IL-17A* rs3819025 SNP.

Genotype Groups	Attribution	n	n (%)	*p*
AA/AG/GG	Chronic pain	9/65/117	4.7/34.0/61.3	0.016 *
	Healthy subjects	29/113/140	10.3/40.1/49.6	
AA/AG + GG	Chronic pain	9/182	4.7/95.3	0.029 *
	Healthy subjects	29/253	10.3/89.7	
AA + AG/GG	Chronic pain	74/117	38.7/61.3	0.013 *
	Healthy subjects	142/140	50.4/49.6	

*: *p* < 0.05.

## Data Availability

The raw data in this study are shown in Datasets S1–S3.

## Supplementary Materials

The following supporting information can be downloaded at: https://www.mdpi.com/article/10.3390/ijms242417627/s1.
