# Supplementary material for: Single-Nucleotide Polymorphisms of the PAR2 and IL-17A Genes Are Significantly Associated with Chronic Pain
Source: Int J Mol Sci. 2023 Dec 18;24(24):17627. doi: 10.3390/ijms242417627 (PMC10744199; doi:10.3390/ijms242417627)
Supplement: Supplementary file 1 [file ijms-24-17627-s001.zip › ijms-2706619-supplementary/Soeda PAR2_IL17A_Figure S1.pptx]

## Slide 1
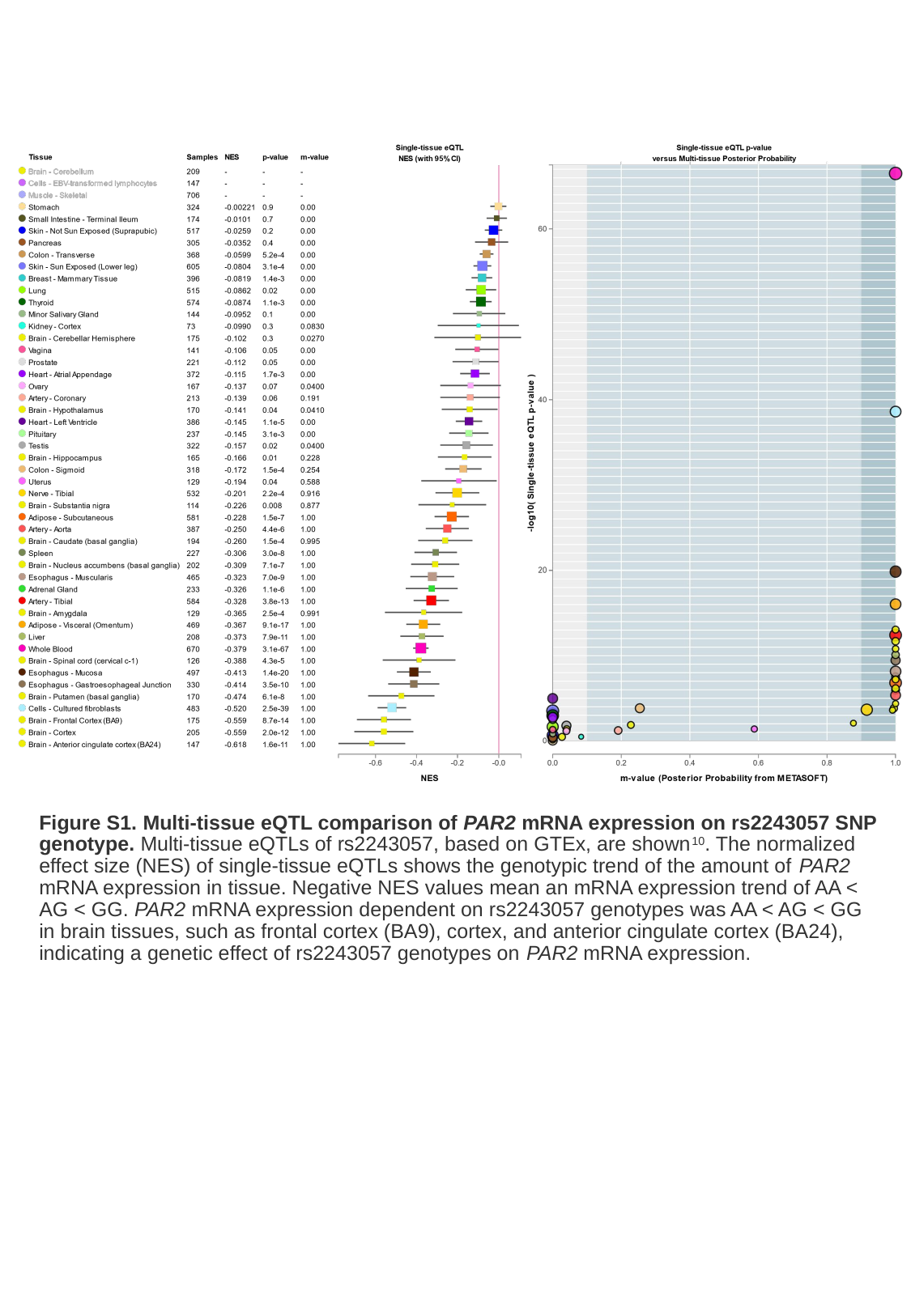

Figure S1. Multi-tissue eQTL comparison of PAR2 mRNA expression on rs2243057 SNP genotype. Multi-tissue eQTLs of rs2243057, based on GTEx, are shown10. The normalized effect size (NES) of single-tissue eQTLs shows the genotypic trend of the amount of PAR2 mRNA expression in tissue. Negative NES values mean an mRNA expression trend of AA < AG < GG. PAR2 mRNA expression dependent on rs2243057 genotypes was AA < AG < GG in brain tissues, such as frontal cortex (BA9), cortex, and anterior cingulate cortex (BA24), indicating a genetic effect of rs2243057 genotypes on PAR2 mRNA expression.
